# Lipomatous Metaplasia in a Warthin Tumor: A Rare Histopathological Finding

**DOI:** 10.1007/s12105-025-01822-x

**Published:** 2025-07-04

**Authors:** Asiye Üzümcü, Melek Büyük, Beyza Nur Baysal, Neslihan Berker, Mine Güllüoğlu

**Affiliations:** Istanbul Faculty of Medicine, Department of Pathology, Istanbul University, Istanbul, Turkey

**Keywords:** Warthin tumor, Lipomatous metaplasia, Parotid gland, Salivary gland, Metaplastic changes, Lipomatous lesion

## Abstract

We present a case of Warthin tumor (WT) exhibiting lipomatous metaplasia. This rare histopathological feature can mimic lipomatous lesions, particularly in hypocellular samples, and should be recognized as a potential diagnostic pitfall. Recognition of this feature is important to avoid diagnostic errors.

## Introduction

WT is the second most common benign salivary gland lesion, typically affecting middle-aged males and showing an increased incidence among smokers [1]. Although metaplastic changes can occur in WT, squamous metaplasia is the most frequently observed type, whereas mucinous metaplasia is considered rare [2].

## Case Report

A 63-year-old male patient presented with a one-year history of a mass located inferior to the right ear. Ultrasonography of parotid glands revealed lobulated solid lesions with cystic degenerative changes, measuring 3.4 × 3 cm on the right and 4.5 × 2.1 cm on the left. Fine-needle aspiration biopsies (FNAB) of both parotid masses were consistent with WT. The patient subsequently underwent right superficial parotidectomy.

**Fig. 1:**
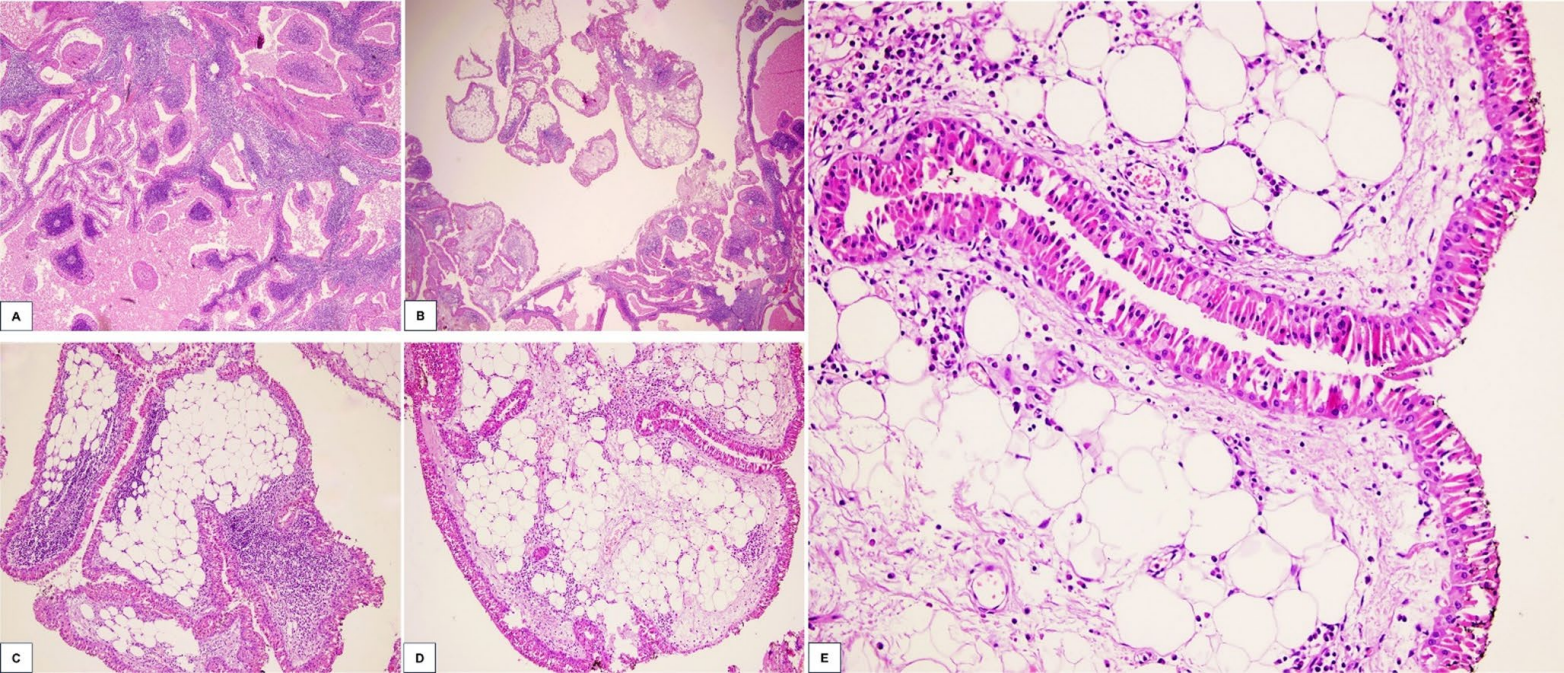
Histologic features of Warthin tumor with lipomatous metaplasia. Warthin tumor shows cystic spaces lined by bilayered oncocytic epithelium and prominent lymphoid stroma (**A**) (H&E, ×100). Foci of mature adipocyte proliferation within the tumor stroma, consistent with lipomatous metaplasia (**B**–**E**) (H&E, ×40, ×200, ×200, ×400, respectively)

On macroscopic examination, the parotid gland measured 8.5 × 6 × 4 cm and contained a nodular, multilobulated, brown-colored mass measuring 4 × 3 × 2 cm, exhibiting hemorrhagic and cystic areas. Histopathological evaluation confirmed the diagnosis of WT, characterized by cystic spaces lined with bilayered oncocytic epithelium and accompanying lymphoid stroma (Fig. 1A). Notably, a 0.4 cm focus of mature adipocyte proliferation, consistent with lipomatous metaplasia, was identified within the tumor (Fig. 1B-E). The possibility of entrapped adipose tissue was considered; however, this was ruled out because the adipose focus was located within the tumor rather than at its periphery. Additionally, the adipose focus was surrounded by bilayered oncocytic epithelium and associated with lymphoid stroma, supporting its intratumoral origin.

## Conclusion

Metaplastic changes in WT are uncommon, with reported incidence rates ranging from 0 to 7.6% in the literature [2]. Squamous metaplasia is the most frequently described subtype and is thought to be associated with prior FNAB [3]. To the best of our knowledge, the lipomatous metaplasia observed in this case represents the first example reported in the literature. The presence of adipocytes in FNAB or core biopsy of salivary glands may lead a diagnostic challenge, potentially resulting in non-diagnostic findings or misinterpretation as a lipomatous neoplasm. Therefore, clinicians and pathologists should be aware that lipomatous metaplasia can occur in WT.

## Data Availability

No datasets were generated or analysed during the current study.

## References

[1] Fisher R, Ronen O (2022) Cytologic diagnosis of parotid gland warthin tumor: systematic review and meta-analysis. Head Neck 44(10):2277–2287. 10.1002/hed.27099 35586869 10.1002/hed.27099 PMC9545504

[2] Yorita K, Nakagawa H, Miyazaki K, Fukuda J, Ito S, Kosai M (2019) Infarcted warthin tumor with mucoepidermoid carcinoma-like metaplasia: a case report and review of the literature. J Med Case Rep 13(1):12. 10.1186/s13256-018-1941-3 30636634 10.1186/s13256-018-1941-3 PMC6330755

[3] Gupta D, Thirunavukkarasu B, Bharti JN, Chugh A, Vishnoi JR (2021) Post fine-needle aspiration near total infarction of warthin tumor with squamous metaplasia: a diagnostic pitfall. Diagn Cytopathol 49(10):1144–1147. 10.1002/dc.24860 34427394 10.1002/dc.24860

